# Combining synchrotron light with laser technology in catalysis research

**DOI:** 10.1107/S1600577518010597

**Published:** 2018-08-23

**Authors:** Sara Blomberg, Johan Zetterberg, Johan Gustafson, Jianfeng Zhou, Mikhail Shipilin, Sebastian Pfaff, Uta Hejral, Per-Anders Carlsson, Olof Gutowski, Florian Bertram, Edvin Lundgren

**Affiliations:** a Synchrotron Radiation Research, Lund University, Box 118, Lund 22100, Sweden; b Combustion Physics, Lund University, Box 118, Lund 22100, Sweden; c Competence Centre for Catalysis, Chalmers University of Technology, Gothenburg 41296, Sweden; d Photon Science, DESY, Notkestrasse 85, Hamburg 22607, Germany

**Keywords:** high-energy surface X-ray diffraction (HESXRD), planar laser-induced fluorescence (PLIF), CO oxidation, Pd(100)

## Abstract

High-energy surface X-ray diffraction (HESXRD) provides surface structural information with high temporal resolution, facilitating the understanding of the surface dynamics and structure of the active phase of catalytic surfaces. The surface structure detected during the reaction is sensitive to the composition of the gas phase close to the catalyst surface, and the catalytic activity of the sample itself may affect the surface structure, which in turn may complicate the assignment of the active phase. For this reason, planar laser-induced fluorescence (PLIF) and HESXRD have been combined during the oxidation of CO over a Pd(100) crystal. PLIF complements the structural studies with an instantaneous two-dimensional image of the CO_2_ gas phase in the vicinity of the active model catalyst. Here the combined HESXRD and PLIF *operando* measurements of CO oxidation over Pd(100) are presented, allowing for an improved assignment of the correlation between sample structure and the CO_2_ distribution above the sample surface with sub-second time resolution.

## Abstract 2

High temporally and spatially resolved *operando* studies of CO oxidation using a Pd(100) single-crystal surface as a model catalyst are presented. The surface structure is determined simultaneously as the gas phase is visualized in two dimensions in the vicinity of the catalyst surface.

## 1. Introduction   

Catalysis is widely used in the industrial production of, for instance, base and speciality chemicals and fuels but is also used in the reduction of toxic emissions from stationary and mobile sources.

The catalyst accelerates a chemical reaction by providing an alternative pathway with a lower-energy barrier for the reaction, without being consumed. The heterogeneously catalyzed pathway often proceeds *via* adsorption of the reactants from a fluid phase onto the surface of a solid catalyst, which mediates the catalytic reaction. A common type of catalyst is the so-called supported catalyst, which often consists of catalytically active nanoparticles embedded in a porous high-surface-area oxide. The complex composition of industrial catalysts and the harsh environmental conditions in the reactor make the feasibility for fundamental surface studies of the catalyst under operating conditions challenging. The details of a catalytic reaction are therefore, in most cases, not fully understood under realistic reaction conditions.

Simplified model systems with, for example, single crystals are therefore used to gain fundamental knowledge about the catalytically active surface (Ertl *et al.*, 1997 ▸). The well defined surface of a single crystal enables the study of specific properties of surface sites or surface structures that are present on the industrial catalytic nanoparticle, and how they contribute to the catalytic activity (Westerström *et al.*, 2007 ▸; Todorova *et al.*, 2003 ▸; Mavrikakis *et al.*, 1998 ▸; Hammer, 2006 ▸; Gustafson *et al.*, 2005 ▸; Gao *et al.*, 2009 ▸; Somorjai & Li, 2010 ▸).

CO oxidation is considered one of the simplest reactions and is therefore traditionally chosen as a model reaction when surface-science studies are performed (Freund *et al.*, 2011 ▸). The experiments are often performed *ex situ* or during exposures at low pressures, typically ranging from 10^−10^ mbar to 10^−6^ mbar. In this manner, the well defined model of the industrial catalyst can be investigated under well controlled conditions and reliable data can be obtained. Despite these previously well controlled *ex situ* studies, the active phase of CO oxidation on a Pd-based catalyst is still under debate (Gao *et al.*, 2009 ▸, 2010 ▸; Rijn, Balmes *et al.*, 2010 ▸). In *operando* studies, the catalyst is studied under working conditions such that the surface structure interacting with gas-phase molecules can be correlated to the function of the catalyst (Blomberg *et al.*, 2013 ▸; Hendriksen *et al.*, 2010 ▸; Ackermann *et al.*, 2005 ▸; Gustafson *et al.*, 2008 ▸; Gao *et al.*, 2009 ▸; Toyoshima & Kondoh, 2015 ▸; Lundgren *et al.*, 2017 ▸; Chen *et al.*, 2007 ▸; Rupprechter & Weilach, 2007 ▸). To achieve a better understanding of the gas–surface interaction, the advantage of having experimental setups which allow the combination of several techniques have been highlighted in the last decade (Head *et al.*, 2017 ▸; Tinnemans *et al.*, 2006 ▸). Some of the advantages of collecting data with several techniques in the same setup are that the results can be achieved under the same conditions. In the ideal case, the techniques can operate simultaneously under operating conditions, giving the opportunity to reach a more detailed understanding of the processes under study.

The activity of the catalyst is often measured by studying the gas composition detected by a mass spectrometer (MS). When the MS is positioned at the outlet of the reactor, as in the present study, the result is a poor spatially resolved measurement and delayed detection of the gas-phase molecules, determined by the path length that the gas molecules travel before reaching the MS detector. For this purpose, we have used planar laser-induced fluorescence (PLIF) to probe the gas phase instantaneously and visualize it close to the catalyst surface (Blomberg *et al.*, 2016 ▸). In the present study, PLIF has been used in combination with high-energy surface X-ray diffraction (HESXRD) for surface structure determination (Gustafson *et al.*, 2014 ▸). By combining these techniques, we can link the surface structure to the CO_2_ production (detected 0.3 mm above the surface non-intrusively) on a sub-second scale. Our observations show that the position of the MS is crucial in determining a correct correlation between catalytic activity and the surface structure of the catalyst.

## 2. Methods   

The experiments were performed at beamline P07 at PETRA III positioned at Deutsches Elektronen-Synchrotron (DESY). This beamline is designed for material studies with hard X-ray radiation (King *et al.*, 2014 ▸). The high-energy X-rays enable full surface-structure determination, where each detector image contains the projection of a large area of the reciprocal space, on a time scale suitable for *operando* studies (Gustafson *et al.*, 2014 ▸; Shipilin *et al.*, 2014 ▸, 2016 ▸; Hejral *et al.*, 2016 ▸, 2017 ▸). The incident angle of the X-rays was set to 0.04°, close to the critical angle of Pd(100) at the photon energy used (85 keV). The diffraction pattern was detected with a temporal resolution of 2 Hz by a two-dimensional Perkin–Elmer detector positioned 1.75 m from the sample. The detector was protected from high-intensity X-rays at the positions of the Pd(100) and the sapphire reactor walls’ Bragg reflections by W pieces. These blocks are seen as dark rectangles in the diffraction images (Fig. 2).

**Figure 1:**
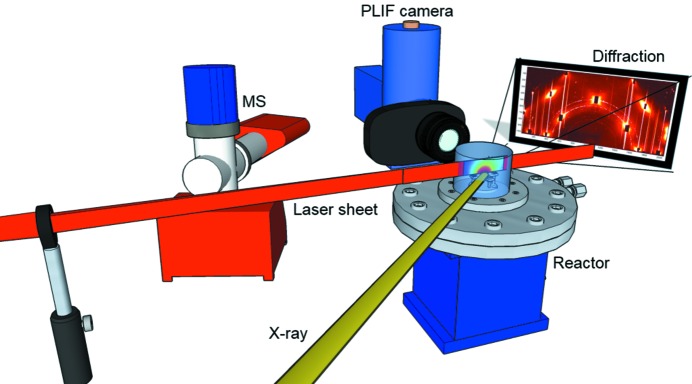
Schematic view of the experimental setup. The diffraction pattern from the (100) surface was detected in the forward direction with respect to the incoming X-rays. The laser sheet, with a wavelength of 2.7 µm, used to probe the CO_2_ gas phase, was sent through the reactor at an angle of approximately 45° relative to the X-rays. The camera for CO_2_ detection was positioned perpendicular to the laser sheet. In addition, an MS was connected to the outlet of the reactor to measure the global gas concentration.

In addition, a laser in the infrared spectral region (2.7 µm) was used to probe CO_2_ in the gas phase in the vicinity (0.3 mm) of the Pd(100) surface at a repetition rate of 10 Hz. PLIF is a species-specific technique, in which the molecule of interest is excited by a laser sheet and then relaxes by emission of a photon (*i.e.* fluorescence), and the fluorescence is detected using a camera. Our target molecule (CO_2_) can be probed *via* ro-vibrational transitions in the mid-infrared. There are several detection schemes that can be employed for probing CO_2_, *e.g.* the overtone and combination band at around 2.0 µm (12^0^1) → (00^0^0) (Alwahabi *et al.*, 2007 ▸), the combination band at 2.7 µm (00^0^0) → (10^0^1) (Zetterberg *et al.*, 2012 ▸; Kirby & Hanson, 2002 ▸) and the fundamental band at around 4.3 µm (Goldenstein *et al.*, 2015 ▸). In this study, the combination band was probed in the CO_2_ molecule using a pulsed laser at ∼2.7 µm with a pulse duration of ∼5 ns and a power of ∼7 mJ pulse^−1^. The laser beam formed a thin laser sheet of ∼6 mm height by sheet-shaping optics and then sent through the sapphire reactor dome ∼0.3 mm above the sample surface. The CO_2_ fluorescence was then imaged onto a two-dimensional focal plane array (FPA) (SBF LP134, Santa Barbara Focal Plane) perpendicular to the laser sheet. The camera exposure time was set to 30 µs and chosen for efficient collection of the CO_2_ fluorescence signal while avoiding detector saturation by the thermal background. To address the varying thermal background, the FPA was triggered at 20 Hz, thus taking an extra image between every laser shot, making subtraction of the thermal background possible on a single-shot basis. A more detailed description of the experimental setup and detection scheme can be found in previous work (Zhou *et al.*, 2017 ▸). Images of the CO_2_ distribution were acquired every 0.1 s, but, for better statistics (signal to noise ratio) in the images and to match the HESXRD repetition rate, the PLIF data were averaged with the result of an image every 0.5 s. Calibration measurements with known CO_2_ partial pressures were performed in order to correlate the detected PLIF signal to partial pressures. A schematic of the setup is shown in in Fig. 1 ▸.

### 2.1. Reactor and sample preparation   

The reactor used for the experiment is based on the same design as the reactor described by Rijn, Ackermann *et al.*, (2010 ▸). For the present experiment, a reactor dome, with a volume of 25 ml, made of sapphire for the transmission of both X-ray and IR wavelengths, was used. Individual gas mass flow controllers (Bronkhorst EL-FLOW) were used for each gas with a capacity to individually flow up to 200 ml_n_ min^−1^. A pressure controller (Bronkhorst EL-PRESS) was attached to the outlet to keep the pressure constant throughout the experiments. The gas composition in the reactor was measured with a quadrupole MS (Pfeiffer PrismaPlus QMG220). The MS was connected to the outlet of the reactor *via* a 4 m-long tube (diameter 6 mm), and a specially made automatic leak valve (LPM Leiden Probe Microscopy BV) was used to regulate the pressure in the MS. An implemented feedback system in the leak valve made it possible to control and keep the pressure constant in the MS, even though drastic changes in the gas flows were applied. The MS data were synchronized with the PLIF images using *LabView* while the HESXRD data and PLIF/MS were synchronized *via* timestamps in their individual data files.

The experimental setup is equipped with an ultra-high vacuum part where cleaning of the sample was carried out. The single crystal was cleaned by Ar^+^ sputtering using an ion energy of 1.5 keV in an Ar pressure of 1 × 10^−5^ Torr. Oxygen treatments removed carbon contaminations by exposing the surface to 1 × 10^−6^ Torr of O_2_ and heating the sample to 900 K.

## 3. Results   

To establish a better picture of the CO oxidation over Pd(100), we performed an *operando* surface-structure determination in combination with CO_2_ gas-phase visualization experiments. HESXRD data images acquired with a photon energy of 85 keV cover a large part of the reciprocal space and reveal the Pd(100) surface structure. Simultaneously recorded PLIF images gained with a laser wavelength of 2.7 µm show the CO_2_ gas-phase distribution above the Pd(100) surface. The surface-structure and gas-phase images were matched, and the CO oxidation could be followed with an updating frequency of 0.5 s. In addition, an MS was positioned at the outlet of the reactor, sampling the global gas concentrations in the reactor.

**Figure 2:**
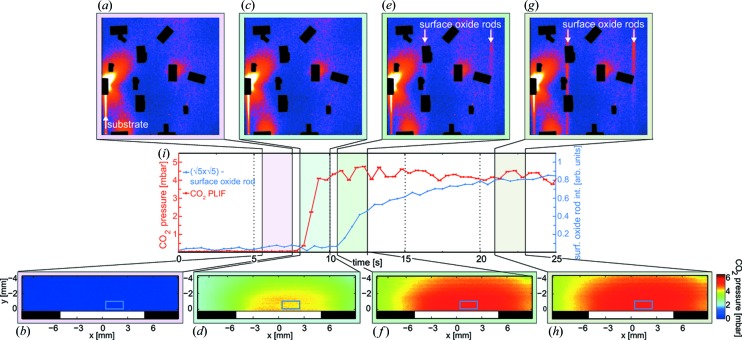
CO oxidation over Pd(100). Each image (HESXRD and PLIF) is averaged over 2.5 s (shown as a colored area in the graph) for better statistics. The graph in (*i*) shows how the CO_2_ PLIF signal, extracted from the area marked with a blue rectangle in (*b*), (*d*), (*f*) and (*h*), together with the intensity of the surface-oxide rod, changes over time. A linear background is subtracted from the plotted HESXRD surface-oxide rod intensity. The data are plotted with an updating frequency of 0.5 s. (*a*) HESXRD image of an inactive sample. A surface truncation rod from the (100) substrate is detected (lower left corner), indicating that a metallic surface is present. (*b*) No CO_2_ is detected in the gas phase above the sample surface. (*c*) A metallic surface is detected in the HESXRD data. (*d*) PLIF image showing the ignition of the reaction as the sample becomes active. (*e*) After the sample has been active for about 2.5 s, superstructure rods from the diffraction of the (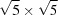)-R27° surface oxide (white arrows) appear in the diffraction pattern. (*f*) As the sample becomes active, a prominent boundary layer of CO_2_ is detected using PLIF. (*g*) After additional reaction time, the (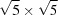)-R27° surface-oxide diffraction pattern intensifies. (*h*) PLIF images showing that the CO_2_ boundary layer is still present over the surface.

To demonstrate the possibility of correlating the surface structure with a change in the gas-phase composition using PLIF and HESXRD, the catalytic activity was modulated by varying the gas ratios of CO and O_2_ at a constant temperature of 200 °C of the sample. The advantage of performing spatially resolved *operando* measurements with high temporal resolution is emphasized in Fig. 2 ▸, where the ignition of the catalyst can be followed in detail over time. At the start of the experiment, we exposed the Pd(100) to 6 mbar of CO and 144 mbar of Ar but no O_2_, which generated an inactive and CO poisoned sample. The diffraction data recorded under this condition (Fig. 2 ▸ *a*) shows Bragg reflections from the bulk and crystal truncation rods from the surface of the Pd(100), demonstrating that a well ordered metallic surface is present when no CO_2_ signal is detected in the gas phase over the surface (Fig. 2 ▸ *b*). To activate the Pd(100) catalyst, the gas composition was changed to reaction conditions by adding 24 mbar of O_2_ simultaneously as the Ar pressure was decreased to 120 mbar in order to maintain a total pressure of 150 mbar constant. Using these reaction conditions, we found that a sample temperature of 200 °C was sufficient to bring the reaction into the mass-transfer limited (MTL) regime where a characteristic boundary layer of CO_2_ was observed (Figs. 2 ▸ *d* and 2*f*), consistent with previous PLIF studies of the gas phase in the MTL regime (Zetterberg *et al.*, 2015 ▸). The boundary layer of the CO_2_ product species accumulated around the surface inhibits the CO molecules reaching the surface and the reaction becomes CO-diffusion limited (Matera & Reuter, 2012 ▸). As a consequence of the boundary layer formation , the gas composition within the boundary layer is significantly different compared with the rest of the reactor, resulting in a more oxidizing environment above the surface in this regime (Blomberg *et al.*, 2016 ▸). At this early stage in the MTL regime, a well ordered oxidized surface is not yet formed and the metallic phase of the surface is still detected in the HESXRD image (Fig. 2 ▸ *c*). We conclude that, approximately 2 s after ignition of the catalyst (Fig. 2 ▸ *e*), a diffraction pattern with surface truncation rods corresponding to the (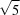 × 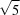)-R27° surface oxide (Todorova *et al.*, 2003 ▸; Gustafson *et al.*, 2014 ▸; Shipilin *et al.*, 2014 ▸) could be observed. After that, the surface oxide rods become more intense and the CO_2_ boundary layer is continuously observed for the remaining time of the experiment.

For the purpose of deactivating the catalyst, the flow of oxygen was turned off, and the same initial conditions of the experiment were present in the reactor. As the CO_2_ PLIF signal dropped, the surface oxide was reduced and could not be observed in the diffraction data when the CO_2_ signal close to the sample was approximately zero.

**Figure 3:**
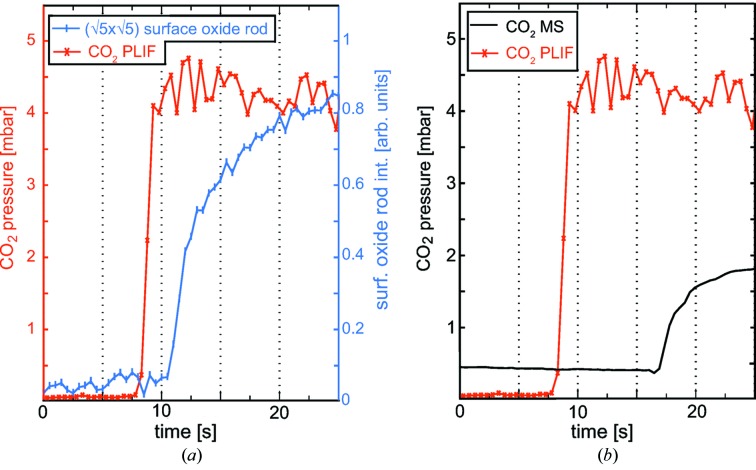
Data extracted from a series of images recorded during the experiment shown in Fig. 2 ▸. (*a*) The total intensity of the superstructure rod from the (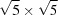)-R27° surface oxide plotted together with the CO_2_ PLIF signal extracted from the blue rectangle indicated in the PLIF images in Fig. 2 ▸, allowing for a better correlation between the surface structure and CO_2_ production. (*b*) The same PLIF CO_2_ signal as in (*a*) plotted together with the CO_2_ MS signal.

To correlate the presence of a surface oxide with the CO_2_ concentration above the catalyst surface, the intensity variation of the truncation rods originating from the (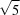 × 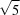)-R27° surface oxide during the CO oxidation experiment, is studied more in detail and compared with the CO_2_ PLIF signal extracted from an area of 1.65 mm^2^, 0.3 mm above the surface (Fig. 3 ▸ *a*). The graph illustrates that the temporal resolution with which we can follow the reaction (HESXRD 2 Hz, PLIF 10 Hz) is sufficient to monitor the ignition of the reaction both in the gas phase and through the surface reconstruction. The sudden increase in CO_2_ production upon sample ignition is easily captured by PLIF though it is clear that the dynamics on the surface forming a well ordered surface oxide is slower. The growth can be followed by studying the intensity of the diffraction rod from the surface oxide, which shows that it takes 2.5 s before any sign of the oxide can be detected. Our results indicate that a metallic surface is present at the ignition of the reaction, but we cannot exclude small domains that are not visible in the diffraction pattern of the (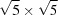)-R27° structure present at the surface. The intensity of the surface-oxide rods increases slowly for around 10 s during the experiment, consistent with a growth of the (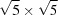)-R27° islands and is interpreted as the surface being, to a large extent, covered with the (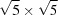)-R27° surface oxide 10 s after ignition.

An MS attached *via* a 4 m-long (diameter 6 mm) gas line at the outlet of the reactor enabled us to achieve information on the global gas concentrations in the reactor. For the purpose of evaluating the gas detecting techniques used in our setup, we compared the MS signal with the PLIF signal (Fig. 3 ▸ *b*). The PLIF signal originates from the CO_2_ gas molecules that are detected almost instantaneously after they desorb from the active surface. Using the PLIF signal as a reference, we determined the response time of the MS to be 9 s. By comparing the PLIF and MS signals, we could also conclude that a CO_2_ partial pressure of around 4–4.5 mbar was measured just above the surface in the MTL regime, while surprisingly, only 2 mbar was detected by the MS during the same period. Neither of the detected CO_2_ concentrations indicate a 100% conversion of CO, but the MS shows a significantly lower concentration than that detected close to the surface using PLIF. The large discrepancy between the measured CO_2_ partial pressures can be explained by the global detection of the gas by MS that suffers from the complex gas-flow configuration in the reactor (Matera *et al.*, 2014 ▸), which smears out the signal as compared with the CO_2_ signal detected just above the surface by PLIF.

## 4. Conclusions and discussion   

The CO oxidation over Pd(100) has previously been studied in great detail, but the question remains whether it is the metallic or oxidized surface that is the most active phase. Herein, we report on the first *operando* measurement where HESXRD, used for surface-structure determination, is combined with PLIF to detect the CO_2_ gas in the vicinity of a catalyst surface. PLIF provides an image of the CO_2_ almost instantaneously as the CO_2_ desorbs from the surface, which enables a correlation to be made between the active surface structure and the gas-phase environment.

PLIF images reveal a boundary layer of CO_2_ close to the surface when the Pd(100) model catalyst is in the MTL regime, resulting in a gas composition in the vicinity of the surface that is significantly different to the concentration detected by the MS at the outlet of the reactor. We conclude that the oxidizing environment, caused by the boundary layer, oxidizes the surface to a large extent, which implies that the catalytic activity of the sample itself affects the surface structure. Our findings show that the surface is not fully oxidized when the ignition of the catalyst occurs, but we cannot exclude that oxidized islands, not detectable with the diffraction, are present on the surface. After several seconds in the highly active regime, however, a strong signal from the surface oxide is detected in the diffraction pattern. Moreover, our data confirm that the response time of the MS is critical when establishing a correlation between surface structure and activity. The delay time of the MS, which is dependent on both the experimental setup and conditions applied, may introduce an uncertainty when measuring and comparing the catalytic activity of a catalyst at different setups, even though identical conditions have been applied. Fortunately, in the present setup, it is possible to measure the response time for the MS by comparing the signals from the MS and PLIF, but to estimate the time delay for the gas detection by MS is generally not trivial. The advantage of using PLIF to complement MS is also demonstrated by extracting the partial pressure of CO_2_, located above the sample surface, which differs significantly from the pressure detected by the MS at the reactor outlet.

In the present study, our measurements were limited by a constant temperature as a result of the critical alignment of the X-rays that should have an incident angle of 0.04^o^ on the sample surface and temperature variation may misalign the sample. It would therefore be interesting to combine PLIF with transmission X-ray diffraction (Reikowski *et al.*, 2017 ▸; Acciarri *et al.*, 1996 ▸), where X-rays penetrate the sample and as a result are less sensitive to temperature variations. The transmission mode of the X-rays would also generate a smaller footprint of the X-ray on the sample surface, which opens up for better spatially resolved surface structure measurements. In the meantime, our results show a unique possibility to relate the activity of the catalyst to the surface structure by combining PLIF and HESXRD. These observations were possible because of the high temporal and spatial resolution in conjunction with immediate detection of desorbed CO_2_ molecules from the active surface.
